# The Effects of Cognitive Therapy versus ‘No Intervention’ for Major Depressive Disorder

**DOI:** 10.1371/journal.pone.0028299

**Published:** 2011-12-09

**Authors:** Janus Christian Jakobsen, Jane Lindschou Hansen, Ole Jakob Storebø, Erik Simonsen, Christian Gluud

**Affiliations:** 1 Psychiatric Research Unit, Copenhagen University Hospital and Region Zealand, Roskilde, Denmark; 2 Copenhagen Trial Unit, Centre for Clinical Intervention Research, Department 3344, Rigshospitalet, Copenhagen University Hospital, Copenhagen, Denmark; Chiba University Center for Forensic Mental Health, Japan

## Abstract

**Background:**

Major depressive disorder afflicts an estimated 17% of individuals during their lifetimes at tremendous suffering and costs. Cognitive therapy may be an effective treatment option for major depressive disorder, but the effects have only had limited assessment in systematic reviews.

**Methods/Principal Findings:**

We used The Cochrane systematic review methodology with meta-analyses and trial sequential analyses of randomized trials comparing the effects of cognitive therapy versus ‘no intervention’ for major depressive disorder. Participants had to be older than 17 years with a primary diagnosis of major depressive disorder to be eligible. Altogether, we included 12 trials randomizing a total of 669 participants. All 12 trials had high risk of bias. Meta-analysis on the Hamilton Rating Scale for Depression showed that cognitive therapy significantly reduced depressive symptoms (four trials; mean difference −3.05 (95% confidence interval (Cl), −5.23 to −0.87; P<0.006)) compared with ‘no intervention’. Trial sequential analysis could not confirm this result. Meta-analysis on the Beck Depression Inventory showed that cognitive therapy significantly reduced depressive symptoms (eight trials; mean difference on −4.86 (95% CI −6.44 to −3.28; P = 0.00001)). Trial sequential analysis on these data confirmed the result. Only a few trials reported on ‘no remission’, suicide inclination, suicide attempts, suicides, and adverse events without significant differences between the compared intervention groups.

**Discussion:**

Cognitive therapy might be an effective treatment for depression measured on Hamilton Rating Scale for Depression and Beck Depression Inventory, but these outcomes may be overestimated due to risks of systematic errors (bias) and random errors (play of chance). Furthermore, the effects of cognitive therapy on no remission, suicidality, adverse events, and quality of life are unclear. There is a need for randomized trials with low risk of bias, low risk of random errors, and longer follow-up assessing both benefits and harms with clinically relevant outcome measures.

## Introduction

According to the WHO, major depressive disorder is the second largest healthcare problem worldwide in terms of illness induced disability [1]. Major depressive disorder afflicts an estimated 17% of individuals during their lifetimes at tremendous cost to the individual and society [2], [3]. Roughly a third of all depressive disorders take a chronic course and, compared to other medical disorders, depressive illnesses causes the most significant deterioration in individual quality of life [4]–[6]. Approximately 15% of all depressive patients will commit suicide over a 10–20 year period [7].

Antidepressant medication remains the mainstay in the treatment of depression [8]. However, meta-analyses have shown that the new antidepressants only obtained beneficial effect in severely depressed patients, and that this effect seems to be clinically small [9], [10]. Antidepressants are, however, known to decrease the risk of relapse [11]. The benefits of antidepressant medication seem to be limited and this raises the question if there are other effective treatments for this serious illness?

Aaron T. Beck originally developed cognitive therapy for depression [12]. Beck believed that critical life events could accentuate hidden negative beliefs, which could generate negative automatic thoughts. These negative thoughts could lead to symptoms of depression, which then could reinforce more negative automatic thoughts. The main goal of the ‘cognitive model of depression’ is to correct these negative beliefs and thoughts, in order to treat the depressive symptoms [12]. A recently published systematic review showed that cognitive therapy might not be an effective treatment for major depressive disorder compared with ‘treatment as usual’ (different forms of non-specific supportive interventions) [13]. Another systematic review shows that cognitive therapy has a preventive effect against recurrent depression, and that this effect clearly surpasses the preventive effects of antidepressant medication [14]. Cognitive therapy versus ‘no intervention’ appears to be an effective treatment for major depressive disorder [15]. We have been unable to find any systematic reviews with meta-analyses, using Cochrane methodology, examining the effect of cognitive therapy versus ‘no intervention’ for major depressive disorder, and the effect size of cognitive therapy is therefore unclear. We embarked on a systematic review using Cochrane methodology to assess the effect of cognitive therapy versus ‘no intervention’ in the treatment of major depressive disorder [16]. We used assessment of bias risk to reduce systematic errors, and trial sequential analysis to reduce the risk of random errors [16]–[19].

## Methods

We conducted our systematic review of randomized clinical trials involving meta-analysis and trial sequential analysis [16]–[19] to answer the question: what are the beneficial and harmful effects of cognitive therapy versus ‘no intervention’ in the treatment of major depressive disorder?

For details regarding the methodology please consult our protocol published on our website (www.ctu.dk) in February 2010, before we began the systematic literature searches in all relevant databases, data-extraction, and analyses [20].

In short, we included all randomized clinical trials comparing the effects of cognitive therapy alone versus ‘no intervention’ alone or cognitive therapy in combination with any co-intervention versus ‘no intervention’ in combination with a similar co-intervention. These co-interventions had to be administered equally in both intervention groups. We did this because we wanted to quantify the effect of cognitive therapy versus ‘no intervention’. No intervention encompassed all potential interventions, including medical treatments; talk therapy; psychology; etc.- except ‘waiting list’. The trials were included irrespective of language, publication status, publication year, and publication type - based on searches in The Cochrane Library's CENTRAL, MEDLINE via PubMed, EMBASE, Psychlit, PsycInfo, and Science Citation Index Expanded (Figure S1. The timeframe for the search was all trials published before February 2010.

To be included, participants had to be older than 17 years with a primary diagnosis of major depressive disorder. Trials were only included if the diagnosis of depression was based on one of the standardized criteria, such as ICD 10 [21], DSM III [22], DSM III-R [23], or DSM IV [24]. Comorbidity with other psychiatric diagnoses was not an exclusion criterion. The following types of trials were excluded:

- Trials focusing on depressed participants with comorbid serious somatic illness, e.g., myocardial infarction, multiple sclerosis, cerebral stroke, cancer, etc.
- Trials focusing on ‘late life’ depression or depression in the elderly, most often participants over 65 years.
- Trials focusing on pregnancy related depression, e.g., postpartum depression, postnatal depression, etc.
- Drug or alcohol dependence related depression.

These exclusions were conducted because we expect participants in such trials to respond differently to standardized psychotherapy than other depressed patients, and these types of depressed patients are traditionally examined in separate trials [25]–[28].

### Interventions

#### Cognitive therapy

Cognitive therapy and cognitive-behavioral therapy are collective terms for a range of different forms of interventions, and it is difficult to find a simple definition which adequately describes this psychotherapeutic method. However, we selected the following criteria from Beck as being necessary for the intervention to be classified as ‘cognitive therapy’ [12]:

1. That the intervention sought to link thoughts, feelings, and behavior, and related these to the depressive symptoms.
2. That the intervention sought to record and correct irrational thoughts or behavioral patterns, and related these to the depressive symptoms.
3. That the intervention sought to teach the patient alternative methods of thinking or behaving, and related these to the depressive symptoms.
4. That the intervention was undertaken face-to-face either individually or in a group.

We accepted any co-intervention to cognitive therapy as long as this co-intervention was similar and administered equally to the experimental group (cognitive therapy) and the control group (‘no intervention’). As mentioned in the introduction, this was done because we wanted to quantify the effect of cognitive therapy.

Furthermore, the trials had to present a treatment manual and had to document adherence to the treatment manual in order for the intervention to be classified as ‘cognitive therapy, adequately defined’. All other trials that classified their intervention as ‘cognitive’ or ‘cognitive-behavioral’ were included, but the intervention was classified under ‘cognitive therapy, not adequately defined’.

### Trial selection

Three of the review authors (JJ, OJS, and JLH) independently selected relevant trials. If a trial was selected by three or two of the three, it was included. If a trial only was identified only by one of the three, it was discussed whether the trial should be included. Excluded trials were entered on a list, stating the reason for exclusion.

### Data extraction

Data were extracted for trial design, bias risk, and outcomes independently by two authors (JJ and JLH). Disagreements were resolved by discussion or through arbitration (CG). We used the instructions in The Cochrane Handbook for Systematic Reviews of Interventions in our evaluation of the methodology and hence bias risk of the trials [16]. We assessed the bias risk in respect to generation of the allocation sequence; allocation concealment; blinding; intention-to-treat analysis; drop-outs; reporting of outcome measures; economic bias; and academic bias. Economic bias may be present if a trial is financed by an individual or organisation that might have an interest in a given result from the trial [16]. Aqcademic bias may be present if one or more of the trialists have an academic or personal interest in a given result from the trial [16]. These components enable classification of the included trials into trials with ‘low risk of bias’ or with ‘high risk of bias’. The trials were overall classified as ‘high risk of bias’ if one or more of the above components was ‘uncertain’ or ‘high risk of bias’ [16], [29]–[32]. This classification is important because trials with ‘high risk of bias’ may overestimate positive intervention effects and underestimate negative intervention effects, and we wanted to relate the validity of our results to the risk of bias in the included trials [16], [29]–[32].

### Primary outcomes

#### Depressive symptoms

Our primary outcomes were the mean value of Hamilton Rating Scale for Depression (HDRS) [33], Beck Depression Inventory (BDI) [34], or Montgomery-Asberg Depression Rating Scale (MADRS) [35] at follow-up. We included data based on the total number of randomized patients (intention-to-treat analysis) if these data were reported. We planned to estimate the therapeutic follow-up responses at two time points:

- At cessation of treatment: The trials' original primary choice of completion date was used. This was the most important outcome measure time point in this review.
- At maximum follow-up.

#### Adverse events

We classified adverse events as serious or non-serious. Serious adverse events were defined as medical events that are life threatening; result in death; disability or significant loss of function; that cause hospital admission or prolonged hospitalization; a hereditary anomaly; or fetal injury [36]. All other adverse events (that is, events that have not necessarily had a causal relationship with the treatment, but that resulted in a change in- or cessation of the treatment) were considered as non-serious events.

#### Quality of life

We included any measure of quality of life noting each assessment measure.

### Secondary outcomes

The proportion of patients not having achieved remission was our first secondary outcome. We included data based on the total number of randomized participants (intention-to-treat analysis) - if possible. If the results were not based on the total number of participants, we preformed an intention-to-treat analysis assuming that the participants not included in the results did not achieve remission [16]. We pragmatically defined remission as a HDRS of less than 8, BDI less than 10, or MADRS less than 10 [34]–[36]. These definitions are also the most commonly used.

Records of suicide inclination, suicide attempts, or suicides were other secondary outcomes.

### Statistical methods

This meta-analysis was undertaken according to the recommendations stated in The Cochrane Handbook for Systematic Reviews of Interventions [16]. In analyzing continuous outcomes with both fixed-effect and with random-effects models, we used the mean difference (MD) with a 95% confidence interval. For statistical calculations we used RevMan version 5.0 [37]. We did not use ‘standardized mean difference’ so each outcome measure was analyzed separately. We did not adjust the outcome variables at follow-up according to the baseline values [16].

We used the odds ratio with a 95% confidence interval to estimate intervention effects on dichotomous outcomes with both fixed-effect and with random-effects models [37].

We performed ‘test of interaction’ [38] for all subgroup analyses [20].

For the primary outcome measure and significant secondary outcome measure, we also conducted trial sequential analysis [39]. In order to calculate the required information size and the cumulative Z-curve's eventual breach of relevant trial sequential monitoring boundaries [16]–[19], the required information size for continuous outcomes was based on a type I error of 5%, a beta of 10% (power of 90%), the variance of all the trials (as no trial had low risk of bias), and a minimal relevant difference of 2 points on the HDRS. For dichotomous outcomes, we based the control proportion on our meta-analytic findings, an assumed relative risk reduction of 30%, a type I error of 5%, a beta of 10% (power of 90%), and the heterogeneity in the meta-analysis.

## Results

### Search results

Our primary literature search identified 4536 publications. According to our protocol [20] we excluded 4137 publications on the basis of the title or abstract, and further 339 citable units were excluded on the basis of the full publication. These exclusions were done either because the publications did not relate to cognitive therapy and depression, or because they were not randomized trials comparing cognitive therapy versus ‘no intervention’. Further 41 publications were excluded because the trial participants or the interventions did not meet our inclusion criteria.

### Included trials

We identified and included 19 publications [40]–[58] on 12 trials [40]–[43], [46]–[51], [53], [55] randomizing a total of 669 participants (Figure S2).

Only six of the trials [40]–[43], [47], [50] used an intervention that we classified as ‘adequately defined’ (see above). We classified the therapists’ level of experience and/or education in two trials as ‘high’ [49], [55], in two trials as ‘intermediate’ [46], [50], in one trials as ‘low’ [40], and in the last seven as ‘unclear’ [41]–[43], [47], [48], [51], [53]. Three trials used cognitive group therapy [40], [42], [51], one trial used a combination of group and individual therapy [53], the remaining eight trials used only individual therapy [41], [43], [46]–[50], [55].

The duration and the extent of the therapy varied in the different trials from six weekly 30 minute sessions of treatment [47] to 24 weeks of treatment (five times a week during the inpatient stay and weekly during the outpatient phase) [55].

**Figure 1:**
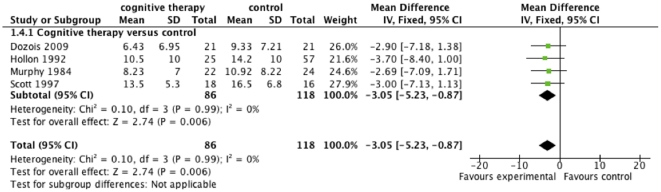
The effect of cognitive therapy versus ‘no intervention’ at cessation of treatment on Hamilton Rating Scale for Depression (HDRS). Below figure: All four trials used only individual cognitive therapy. The therapists' level of experience and/or education was classified as ‘high’ in Dozios (2009), as ‘intermediate’ in Murphy (1984) and Hollon (1992), and as ‘unclear’ in Scott (1997).

Eight trials used the experimental intervention cognitive therapy as add on therapy to antidepressant medicine [46]–[51], [53], [55]. All of the eight trials used different antidepressants (Figure 1). The antidepressant medicine was delivered similarly in the experimental and control groups in all of the trials.

Blackburn et al. (1981) examined the effect of cognitive therapy and antidepressants versus antidepressants [51]. The participants were assessed with HDRS and BDI. The results at the end of treatment show a significant effect of cognitive therapy compared with the control. However, the trial did not report SD for the mean values.

Teasdale et al. (1984) examined the effect of cognitive therapy and ‘treatment as usual’ versus ‘treatment as usual’ [41]. The participants were assessed with HDRS, BDI and MADRS. However, the results were only reported as median values. Participants receiving cognitive therapy had improved significantly greater than the control group on all three scales at cessation of treatment. There was no significant difference at three months follow-up.

Usaf et al. (1990) examined the effect of cognitive therapy versus ‘waiting list’ [42]. The participants were assessed with BDI. Participants receiving cognitive therapy had a non-significant greater improvement on outcome measures compared with control. However, the trial did not report SD for the mean values

Wright et al. (2005) examined the effect of cognitive therapy versus ‘waiting list’ [43]. The participants were assessed with HDRS and BDI. The results at end of treatment showed a significant effect of cognitive therapy compared with control. However, the trial did not report mean values and SD. The authors chose to report change in scores instead of mean on continuous outcome, because the baseline-means were significantly different between the two intervention groups.

We have written to the authors of the four trials in the above [41]–[43], [51] requesting the necessary data. We have received no answer so we have not been able to include the data from these four trials in the following analysis.

Table 1 summarizes the characteristics of the 12 included trials.

**Table 1:** Characteristics of the included trials.

Trial	Particiants (randomized)	Interventions	Outcomes and notes
Blackburn 1981	42	Cognitive therapy (individual, 20 weeks)+150 mg amitriptyline or 150 mg clomipramine	HDRS, BDINo means or SD
Murphy 1984	46	Cognitive therapy (individual, 12 weeks)+nortriptyline (TCA) versus nortriptyline (TCA) dose of nortriptyline: 50 to 150 ng in venous blood	HDRS, BDI, remission (HDRS<8, HDRS<7) and BDI<10)
Teasdale 1984	44	Cognitive therapy (individual, 20 sessions)+‘treatment as usual’ versus ‘treatment as usual’‘treatment as usual’: general practitioners were asked to treat patients as they would normally	HDRS, BDI and MADRS.No means and SD (report median scores)
Ross 1985	67	Cognitive therapy (individual and group, 12 weeks)+‘treatment as usual’ versus waiting list+‘treatment as usual’‘treatment as usual’: treatment by the referring GP including different antidepressants	BDI and Montgomery- Asberg scale.10/67 of the participants had only ‘probable major depressive disorder
Miller 1989	32	Cognitive therapy (5 weekly individual sessions for 4 weeks followed by 1 weekly session for 20 weeks)+‘standard treatment’ versus ‘standard treatment’‘standard treatment’ included use of antidepressants (amitriptyline and desipramine)	BDI, Modified HDRS Scale for Suicidal Ideation and remission (BDI<10, HDRS<7)Participants were inpatients
Usaf 1990	60	Cognitive therapy (group, 8 weeks) versus ‘waiting list’	BDI.No means or SD
Hollon 1992	82	Cognitive therapy (individual, 12 weeks)+75–300 mg imipramine versus 75–300 mg imipramine	HDRS, BDI, Raskin Depression Scale and remission (HDRS<7, BDI<10)
Scott 1997	48	Cognitive therapy (individual, 6 weeks)+‘treatment as usual’ versus ‘treatment as usual’‘treatment as usual’: treatment by GP including different antidepressants	HDRS and BDI
Wright 2005	30	Cognitive therapy (individual, 8 weeks) versus ‘waiting list’	HDRS and BDI.No SD
Shamsaei 2008	80	Cognitive therapy (individual, 8 weeks)+citalopram (SSRI) versus citalopram (SSRI)	BDI
Wong 2008	96	Cognitive therapy (group, 10 weeks) versus waiting list	BDI (Chinese BDI)
Dozois 2009	42	Cognitive therapy (individual, 15 weeks)+antidepressants versus antidepressants antidepressants: SSRI, SNRI and TCA	HDRS, BDI

### Bias risk

We assessed all of the 12 included trials [40]–[43], [46]–[51], [53], [55] as having ‘high risk of bias’ due to unclear or inadequate components as described in Table 2.

**Table 2:** Risk of bias.

	Allocation sequence generation?	Allocation concealment?	Intention to treat analysis?	Blinding?	Comparability of drop-outs in intervention groups?	Free of selective outcome measure reporting?	Free of economic bias?	Free of academic bias?	Overall bias assessment
Blackburn 1981	Unclear	Unclear	No	Unclear	Yes	Unclear	Unclear	Unclear	High risk of bias
Murphy 1984	Yes	No	Yes	No	Unclear	Unclear	Yes	Unclear	High risk of bias
Teasdale 1974	Unclear	Unclear	No	Yes	No	Unclear	Yes	Unclear	High risk of bias
Ross 1985	Yes	Unclear	Yes	Yes	Unclear	Unclear	Unclear	Unclear	High risk of bias
Miller 1989	Unclear	Unclear	No	No	Yes	Unclear	Unclear	Unclear	High risk of bias
Usaf 1990	Unclear	Unclear	No	No	No	Unclear	Unclear	Unclear	High risk of bias
Hollon 1992	Unclear	Unclear	No	Yes	Yes	Unclear	Unclear	Unclear	High risk of bias
Scott 1997	Unclear	Unclear	No	Unclear	No	Unclear	Yes	Unclear	High risk of bias
Wright 2005	Unclear	Unclear	Yes	Yes	Yes	Unclear	Unclear	Unclear	High risk of bias
Shamsaei 2008	Yes	Unclear	Unclear	Unclear	Unclear	Unclear	Yes	Unclear	High risk of bias
Wong 2008	No	Unclear	Unclear	Unclear	No	Unclear	Unclear	Unclear	High risk of bias
Dozois 2009	Unclear	Unclear	No	Unclear	Yes	Unclear	Unclear	Unclear	High risk of bias

### Primary outcome measures

#### Depressive symptoms

Four trials assessed and reported HDRS as a continuous outcome measure at the end of treatment [46], [47], [49], [50]. Eight trials also assessed and reported BDI [40], [46]–[50], [53], [55].

Meta-analysis with the fixed-effect model on the HDRS data from the four trials [46], [47], [49], [50], shows that cognitive therapy at the end of therapy significantly reduced depressive symptoms compared with ‘no intervention’. We found a mean difference on −3.05 HDRS (95% CI −5.23 to −0.87; P<0.006, I^2^ = 0) (Figure 1). The I^2^ statistic describes the percentage of variation across trials that are due to heterogeneity rather than chance. Meta-analysis with the random-effects model gave identical results.

**Figure 2:**
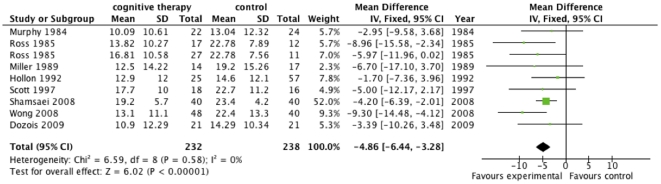
The effect of cognitive therapy versus ‘no intervention’ at cessation of treatment on Becks Depression Inventory (BDI).

Meta-analysis with the fixed-effect model on the BDI data from the eight trials [40], [46]–[50], [53], [55] was in agreement with the results from HDRS (mean difference on −4.86 BDI (95% CI −6.44 to −3.28; P = 0.00001, I^2^ = 0)) (Figure 2). Meta-analysis with the random-effects gave identical results.

**Figure 3:**
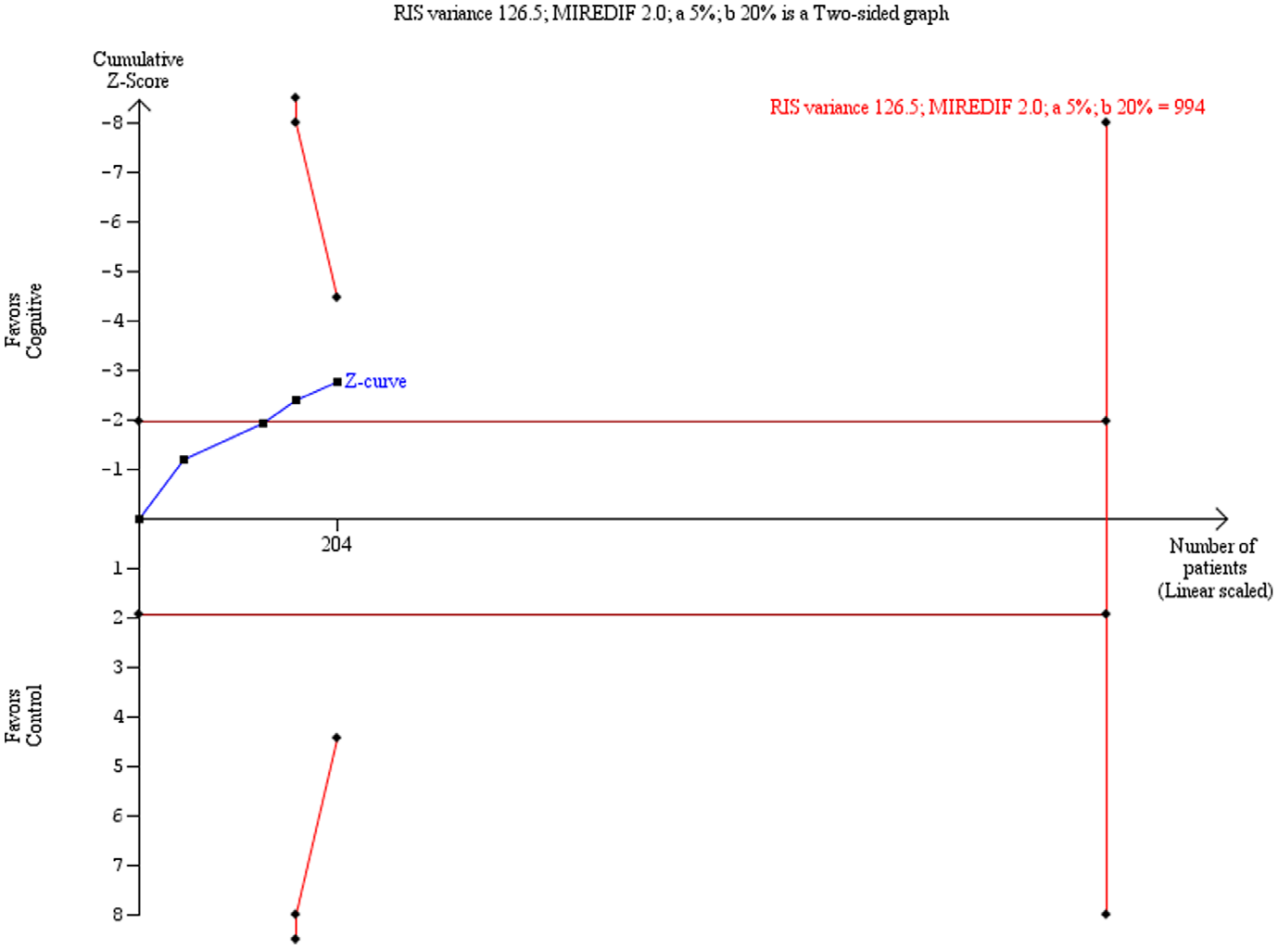
Trial sequential analysis of the cumulative meta-analysis of the effect of cognitive therapy versus ‘no intervention’ for major depressive disorder on the Hamilton Rating Scale for Depression (HDRS). Below figure: The required information size of 994 is calculated based on an intervention effect compared with ‘no intervention’, of 2 points on the HDRS, a variance of 126.5.04 on the mean difference, a risk of type I error of 5%, and a power of 80%. With these presumptions, the cumulated Z-curve (blue curve) do not cross the trial sequential monitoring boundaries (red inner sloping lines) implying that there is no firm evidence for a beneficial effect of cognitive therapy compared with no intervention.

**Figure 4:**
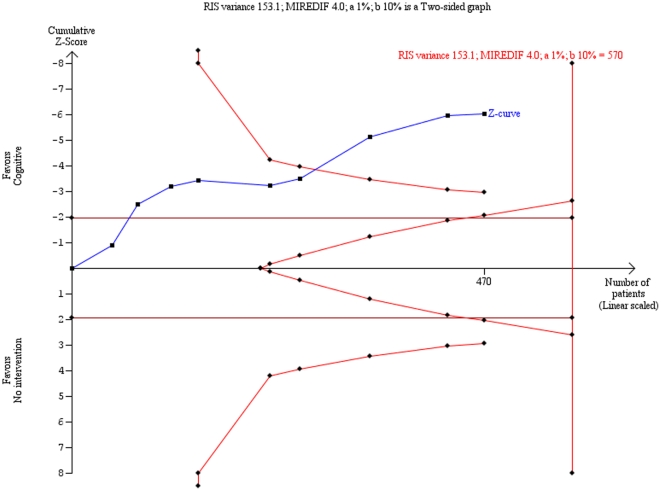
Trial sequential analysis of the cumulative meta-analysis of the effect of cognitive therapy versus no ‘intervention’ for major depressive disorder on the Beck Depression Inventory (BDI). Below figure: The required information size of 570 is calculated based on an intervention effect compared with ‘no intervention’, of 4 points on the BDI, a variance of 153.1 on the mean difference, a risk of type I error of 1% and a power of 90%. With these presumptions, the cumulated Z-curve (blue curve) crosses the trial sequential monitoring boundaries (red inner sloping lines) implying that there is no risk of random error in the estimate of a beneficial effect of cognitive therapy compared with no intervention. However, all trials were considered as high risk of bias, which could explain the positive findings.

Trial sequential analysis on the HDRS data showed that ‘insufficient data’ have been obtained to decide if cognitive therapy is superior compared with ‘no intervention’ (Figure 3). Trial sequential analysis on the BDI data showed a significant beneficial effect of cognitive therapy compared with ‘no intervention’ (Figure 4).

Only two of the trials included assessment data after the cessation of treatment on the HRDS [46], [47]. Murphy et al. (1984) assessed the participants at one month after cessation of treatment and Scott et al. (1997) at one year after cessation of treatment [46], [47]. Meta-analysis with fixed-effect model on these data showed a mean difference on −0.32 HDRS points (95% CI −0.85 to −0.22; P = 0.25, I^2^ = 57%) and −3.68 BDI points (95% CI −8.11 to −0.75; P = 0.10, I^2^ = 0) in favor of cognitive therapy. Meta-analysis with random-effects gave an identical result.

#### Adverse events

Two trials reported adverse events [40], [50]. Hollon et al. (1992) reported five serious adverse events in the control group (two participants hospitalized due to symptomatic worsening and three experiencing severe adverse reactions to concomitant medications) [50]. Wong et al. (2008) reported one hospitalization in the control group [40]. None of the remaining trials reported on adverse events.

#### Quality of life

None of the included trials assessed the quality of life of the participants.

### Secondary outcome measures

#### Participants without remission

Three trials reported the proportion of participants without remission as a dichotomous outcome measure [46], [50], [55]. We had planned to define remission as a Hamilton score of less than 8, BDI less than 10, or MADRS less than 10. However, this was not possible, so we adopted the slightly different definitions of the individual trials. All three trials defined remission as HRDS less than 7 [46], [50], [55], while one trial also defined remission as a Hamilton score of less than 8 [46]. All three trials also defined remission as BDI less than 10 [46], [50], [55].

**Figure 5:**
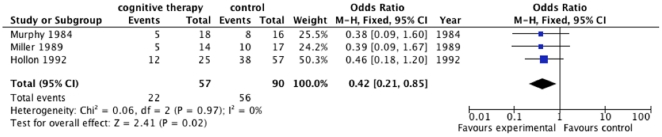
Effect of cognitive therapy versus ‘no intervention’ on ‘no remission’ (HDRS>7) at cessation of treatment.

**Figure 6:**
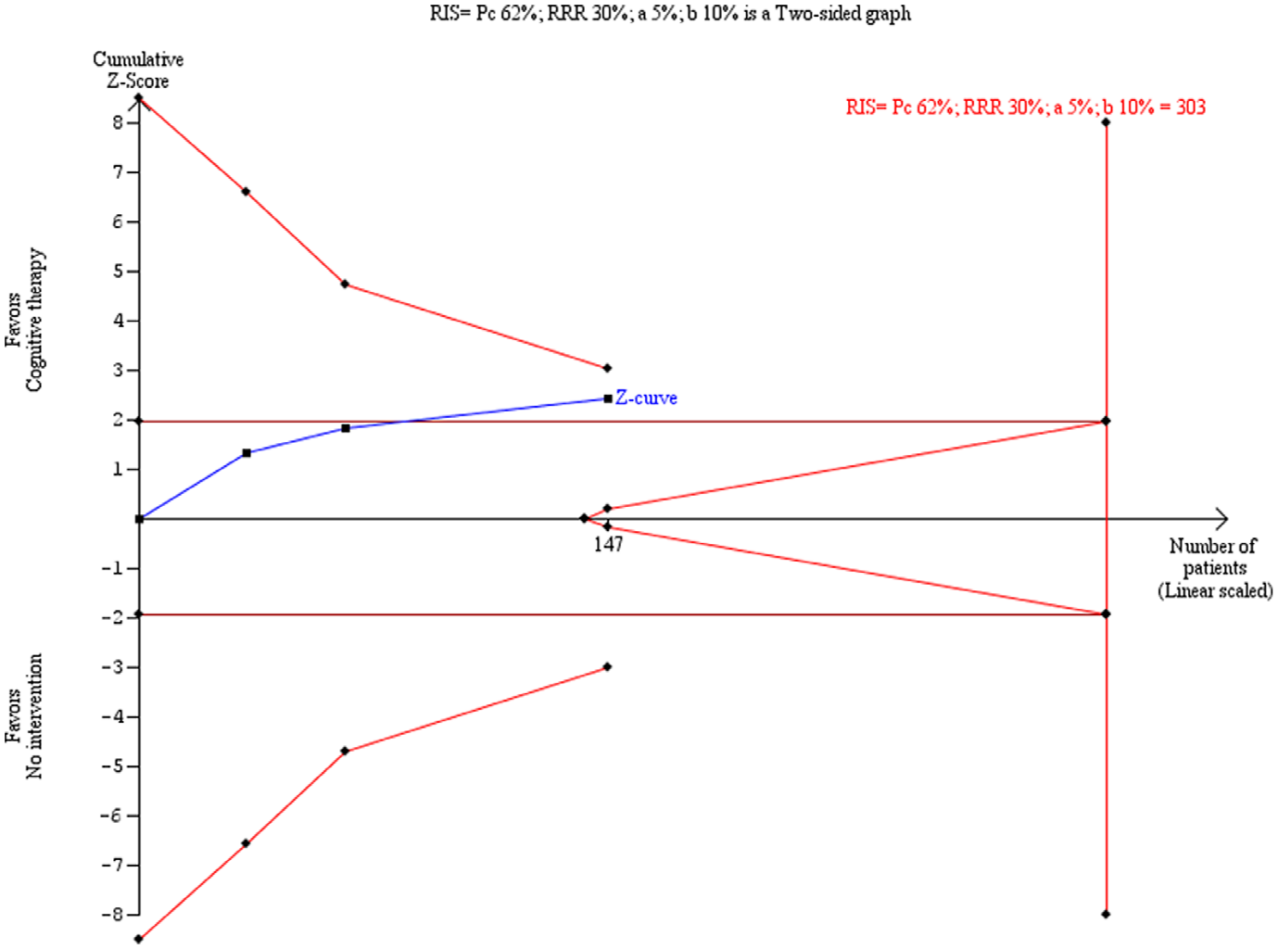
Trial sequential analysis of the cumulative meta-analysis of the effect of cognitive therapy versus no ‘intervention’ for no remission according to the Hamilton Rating Scale for Depression. Below figure: The required information size of 303 is calculated based on a control event proportion of 62%, an assumed relative risk reduction of 30%, a type I error of 5%, a beta of 10% (power of 90%), and the heterogeneity in the meta-analysis. With these presumptions, the cumulated Z-curve (blue curve) do not cross the trial sequential monitoring boundaries (red inner sloping lines) implying that there is a risk of random error in the estimate of a beneficial effect of cognitive therapy compared with no intervention, either due to sparse data or repetitive testing in the cumulative meta-analysis. Furthermore, all trials were considered as high risk of bias, which could explain the positive findings in the conventional meta-analysis.

Meta-analysis on the HDRS data from the three trials [46], [50], [55] showed that cognitive therapy compared with ‘no intervention’ significantly decreases the risk of ‘no remission’ with an odds ratio of 0.42 (95% CI, 0.21 to 0.85; P = 0.02, I^2^ = 0) (Figure 5). The number needed to treat to obtain one extra patient with remission is about four patients (95% CI, 3 to 13). Trial sequential analysis on these data shows that we cannot exclude risk of random errors due to sparse data and repetitive testing as the cause for the meta-analysis result (Figure 6).

The meta-analysis on the BDI-data from the three trials [46], [50], [55] showed that cognitive therapy compared with ‘no intervention’ did not significantly decrease the risk of ‘no remission’ with an odds ratio of 0.54 (95% CI, 0.27 to 1.09; P = 0.08, I^2^ = 0).

#### Participants with suicidal inclination

Teasdale et al. (1984) reported numbers of patients that deliberate self-poisoned [41]. No patient in the cognitive therapy group self-poisoned. Two of the patients in the control group were treated for deliberate self-poisoning.

Miller et al. (1989) trial used the Modified Scale for Suicidal Ideation [55]. They found no significant difference in suicidal ideation between the different intervention groups, and recorded no suicide attempts or suicides during the trial period.

Hollon et al. (1992) reported three suicide attempts [50], one participant randomized to cognitive therapy and two participants randomized to the control intervention. One from each group died from their attempt.

Wong et al. (2008) reported no suicide attempts in the cognitive therapy group and two suicide attempts in the control group during the intervention period [40]. Neither of these participants died from their attempt.

None of the remaining trials included records of suicide inclination, suicide attempts, or suicides.

### Subgroup analyses

According to our protocol [20] we had planned a number of subgroup analyzes, but we found no heterogeneity in our results. We therefore did not conduct subgroup analyses of therapists' level of education and experience (high versus intermediate versus low versus unclear), type of therapy (group versus individual), and use of antidepressants as co-intervention (antidepressant co-intervention versus no antidepressant co-intervention). Our findings indicate that these factors do not seem to influence the effect of cognitive therapy.

We had also planned a subgroup-analysis according to risk of bias [20]. However, as all trials were classified as ‘high risk of bias’ it was not possible to conduct this analysis.

## Discussion

The results of our systematic review with meta-analysis and trial sequential analysis (on the BDI-data) suggest that cognitive therapy may significantly reduce depressive symptoms on the HDRS and BDI, and may increase the probability of remission compared with ‘no intervention’. The number needed to treat to obtain one extra patient with remission is about four patients (95% CI, 3 to 13), but trial sequential analysis suggests that this result could be due to random error due to sparse data or repetitive testing (NY ref). When evaluating these data, one should notice that all trials were considered to have high risks of bias which could lead to overestimation of beneficial intervention effects.

The present review has a number of strengths. Our protocol [20] was published before we began systematic literature searches in all relevant databases, data extraction, and data analysis. Data was extracted by two independent authors minimizing the risk of inaccurate data-extraction, and we assessed the risk of bias in all trials according to the Cochrane Handbook for Systematic Reviews of Interventions [16]. We meta-analyzed data both with fixed-effect and random-effects models and both analyses were in agreement in all our results. Furthermore, we performed trial sequential analysis to assess the risk of random errors [16]–[19]. The results of the trial sequential analysis on the BDI data confirmed the cumulative meta-analysis result. Trial sequential analysis on the HDRS data showed that insufficient data have been obtained, but this analysis was based on the results from only four trials. The trial sequential analysis result also indicates that in order to detect or reject an intervention effect with a minimal relevant difference of two points on HDRS, a required information size of 994 participants may be needed. With a relatively limited number of trials and trial participants and with an increasing number of repetitive tests, the risk of falsely rejecting the null hypothesis (type I error) is substantial Trial sequential analysis is a statistical analysis that enables one to assess the risks of random errors that may occur due to sparse data and multiple testing on accumulating data. Trial sequential analysis is therefore a more robust analysis than the traditional cumulative meta-analysis [16]–[19].

Our systematic review has a number of limitations. The characteristics of the participants in the different trials, as well as the severity of the depressive symptoms differed. E.g., the participants in Miller et al. (1989) were inpatients and Scott et al. (1997) examined the effect of cognitive therapy for depression in primary care patients [47], [55]. Nine [41], [46]–[51], [53], [55] of the 12 included trials used some form of antidepressants as co-intervention to cognitive therapy. We did not, however, find any heterogeneity in our analyses and although head-to-head comparisons are needed in order to thoroughly examine differences between intervention groups, this indicates that there is a comparable treatment effect regardless of the use of antidepressants as co-intervention and among the different populations treated. This may make our results more generally applicable. On the other hand, only few trials with few participants were included, which may decrease the external validity of our results.

The fact that we were only able to include 12 trials with a limited number of participants also raises other concerns. Only four of the 12 trials reported mean and SD for HDRS, and only 8 of the 12 trials reported means and SD for BDI. None of the included trials were assessed as being free of ‘selective outcome measure reporting bias’ [16]. There is therefore a risk of within-study selective outcome reporting in the 12 trials. Furthermore, all 12 trials had an overall assessment as ‘high risk of bias’ - so our results may be questionable. Moreover, trial sequential analysis on the effect on HDRS showed that we could not exclude the risk of random errors [16]–[19]. Due to the limited number of included trials we did not perform a funnel plot or other analysis to explore the risk of publication bias [16]. Other meta-analyses have shown that publication bias significantly has influenced the results from former publications [9]. It is a further limitation that we are not able to assess the risk of publication bias.

Only six of the trials used an intervention that we classified as ‘adequately defined’, i.e., using and documenting the use of a therapeutic manual. And although we did not find any heterogeneity in our results it is imperative in clinical trials that the interventions are adequately defined and described [59]. Factors like personal style, communication skills, and personality of the therapist evidently will influence the way psychotherapy is delivered [60], and it is difficult to describe and control for these subjective factors. It is therefore important to relate psychotherapeutic interventions to a treatment manual. Otherwise it is unclear what kind of intervention the participants were receiving, and it is difficult to apply any result in clinical practice.

A number of subgroups of depressed patients were not included in the trials of this review. These subgroups may react differently to psychotherapy and of course our review cannot be generalized to other than the included patient groups.

Because we wanted to quantify the effect of cognitive therapy, any co-intervention had to be delivered similarly in the experimental intervention group and the control group. So forth, our results show that the benefit from this relatively extensive treatment compared with ‘no intervention’ was only a few points on HDRS and BDI. We believe that these mean differences are relatively small from a clinical viewpoint - especially if you relate these mean differences to the extent and length of the intervention. On the other hand, our analyses demonstrate that the number needed to treat to obtain one extra patient in remission was only about four patients. This estimate was based on only three trials, which primarily defined remission as a HDRS or a BDI score under a given value. Again, we are not able to exclude the risks of systematic errors (bias) and random errors (play of chance) on this estimate.

Depression is generally a difficult condition to treat effectively and other reviews have found similar effects for other forms of psychotherapy [61]. However, the HDRS might not be a useful instrument to quantify the effect of cognitive therapy. Other assessment methods could demonstrate a more substantial effect of any given intervention for depression. Furthermore, severity of depression as measured by the total HDRS score has failed to predict suicide attempts [62], and some publications have questioned the usefulness of the HDRS and concluded that the scale is psychometrically and conceptually flawed [63]. The two other outcome measures often used to assess depressive symptoms, MADRS and BDI, probably correspond to HDRS [64], [65]. The HDRS has during 40 years been the gold standard to quantify depressive symptoms in clinical trials [63]. There may be a need for other assessment methods.

Only two of the trials included assessments after the cessation of treatment. Therefore it is not clear whether cognitive therapy has any effect on depressive symptoms in the longer term.

None of the trials reported measures of quality of life. Outcome measures of quality of life are generally not standardized and thoroughly individually validated [69]. The use of standardized outcome measures for quality of life in research has been limited by difficulties in administering and scoring quality of life, but quality of life can be used as a valid outcome measure [31], [66].

Only two of the included trials reported on some adverse events and only four of the included trials included some records of suicide inclination, suicide attempts, or suicides. Typically adverse events are not reported as thoroughly as beneficial outcome measures [67]. Some psychological interventions might have harmful effects. E.g., psychological debriefing for preventing post-traumatic stress disorder has in some clinical trials showed to have a harmful effect [68]. Possible harmful effects of cognitive therapy are not thoroughly examined.

Future research should focus on comparing different forms of manualized psychotherapy and comparing cognitive therapy with other treatments for depression. First and foremost such trials should be conducted with lower risk of bias (‘systematic errors’) and lower risk of random errors (‘play of chance’) as well as longer follow up [69]. Such trials should also report on adverse events, suicide inclination, suicide attempts, and numbers of suicides. There may also be a need for a new gold standard assessment method other than HRDS to assess depressive symptoms, and if possible more effective interventions for depression must be developed. But first and foremost trials with lower risk of bias and lower risk of random errors are needed.

### Conclusions

Cognitive therapy might be an effective treatment for depression measured on HDRS and BDI, but the effects on suicidality, adverse events, and quality of life are unclear. There is a need for randomized trials with low risk of bias, low risk of random errors, and longer follow-up assessing both benefits and harms with clinically relevant outcome measures.

## Supporting Information

Figure S1 **‘Search strategy’.**
(PDF)

Figure S2 **‘PRISMA Flowchart’.**
(TIF)
