# Supplementary material for: The Effects of Cognitive Therapy versus ‘No Intervention’ for Major Depressive Disorder
Source: PLoS One. 2011 Dec 9;6(12):e28299. doi: 10.1371/journal.pone.0028299 (PMC3235113; doi:10.1371/journal.pone.0028299)
Supplement: Figure S1 — ‘Search strategy’. (PDF) [file pone.0028299.s001.pdf]

Cochrane library -

#1 MeSH descriptor Depressive Disorder, Major explode all trees
#2 MeSH descriptor Depressive Disorder, this term only
#3 MeSH descriptor Seasonal Affective Disorder explode all trees
#4 MeSH descriptor Dysthymic Disorder explode all trees
#5 MeSH descriptor Depression explode all trees
#6 MeSH descriptor Affective Symptoms, this term only
#7 (#1 OR #2 OR #3 OR #4 OR #5 OR #6)
#8 MeSH descriptor Cognitive Therapy explode all trees
#9 (#7 AND #8)

MEDLINE

1 depressive disorder/ or depressive disorder, major/
or dysthymic disorder/ or seasonal affective disorder/
2 exp Depression/
3 exp Affective Symptoms/
4 1 or 3 or 2
5 randomized-controlled-trial.pt.
6 controlled clinical trial.pt.
7 randomized controlled trials.mp.
[mp=title, original title, abstract, name of substance word, subject heading word]
8 random allocation.mp.
[mp=title, original title, abstract, name of substance word, subject heading word]
9 double blind method.mp.
[mp=title, original title, abstract, name of substance word, subject heading word]
10 single blind method.mp.
[mp=title, original title, abstract, name of substance word, subject heading word]
11 clinical trial.pt.
12 (clin$ adj25 trial$).ti,ab.
13 ((singl$ or doubl$ or tripl$ or trebl$) adj25
(blind$ or mask$ or dummy$)).mp.
14 exp clinical trial/
15 placebos.mp. [mp=title, original title, abstract,
name of substance word, subject heading word]
16 placebo$.ti,ab.
17 random$.ti,ab.
18 comparative study.mp. [mp=title, original title, abstract,
name of substance word, subject heading word]
19 evaluation studies as topic/ or exp clinical trials as topic/
20 follow up studies.mp. [mp=title, original title, abstract,
name of substance word, subject heading word]
21 prospective studies.mp.
[mp=title, original title, abstract, name of substance word, subject heading word]
22 (control$ or prospectiv$ or volunteer$).ti,ab.
23 11 or 21 or 7 or 17 or 22 or 18 or 16 or 13 or 6 or 9

or 12 or 14 or 15 or 20 or 8 or 10 or 19 or 5

24 4 and 23
25 exp cognitive therapy/ or exp sensitivity training groups/
26 25 and 24

EMBASE

1 depression/
2 controlled study.de.
3 clinical trial.de.
4 major clinical study.de.
5 randomized controlled trial.de.
6 double blind procedure.de.
7 clinical article.de.
8 random$.mp.
9 control$.mp.
10 follow up.mp.
11 ((singl$ or doubl$ or tripl$ or trebl$) adj (blind$ or mask$ or dummy)).mp.
12 placebo$.mp.
13 (clinic$ adj (trial$ or study or studies$)).mp.
14 exp comparative study/
15 6 or 11 or 3 or 7 or 9 or 12 or 2 or 14 or 8 or 4 or 13 or 10 or 5
16 1 and 15
17 Cognitive Therapy/
18 16 and 17

PsycINFO

1 exp affective disturbances/
2 exp major depression/
3 exp "depression (emotion)"/
4 exp dysthymic disorder/
5 exp anaclitic depression/
6 exp endogenous depression/
7 exp atypical depression/
8 exp reactive depression/
9 exp recurrent depression/
10 exp treatment resistant depression/
11 6 or 3 or 7 or 9 or 2 or 8 or 1 or 4 or 10 or 5
12 random$.mp.
13 ((singl$ or doubl$ or trebl$ or tripl$) adj25 (blind$ or dummy or mask$)).mp.
14 placebo$.mp.
15 crossover.mp.
16 assign$.mp.
17 allocat$.mp.
18 ((clin$ or control$ or compar$ or evaluat$ or prospectiv$) adj25 (trial$ or studi$ or study)).mp.
19 exp placebo/

20 exp treatment effectiveness evaluation/
21 exp mental health program evaluation/
22 exp experimental design/
23 versus.id.
24 vs.id.
25 21 or 17 or 12 or 20 or 15 or 14 or 22 or 18 or 24 or 23 or 13 or 16 or 19
26 25 and 11
27 exp cognitive therapy/ or exp cognitive behavior therapy/
28 27 and 26

CINAHL

S19 S18 and S17
S18 (MH "Cognitive Therapy") or
(MH "Cognitive Therapy (Iowa NIC) (Non-Cinahl)+")
S17 S16 and S6
S16 S15 or S14 or S13 or S12 or S11 or S10 or S9 or S8 or S7
S15 TX control* OR prospectiv* OR volunteer*
S14 MH Prospective Studies
S13 MH Evaluation Research+
S12 TX placebo* OR random*
S11 MH Placebos
S10 TX clin* N25 trial*
S9 TX (singl* OR doubl* OR tripl* OR trebl*) AND
(blind* OR mask* OR dummy*)
S8 PT clinical trial
S7 MH Clinical Trials+
S6 S5 or S4 or S3 or S2 or S1
S5 MH Affective Symptoms
S4 MH Affective Disorders
S3 MH Dysthymic Disorder
S2 MH depression, reactive
S1 MH depression

AMED

S17 S16 and S15
S16 (DE "COGNITIVE THERAPY")
S15 S14 and S3 (688)
S14 S13 or S12 or S11 or S10 or S9 or S8 or S7 or S6 or S5 or S4
S13 TX (clin* or control* or compar* or evaluat* or prospectiv*)
AND (trial* or studi* or study)
S12 TX (singl* OR doubl* OR tripl* OR trebl*) AND
(blind* OR dummy OR mask*)
S11 TX allocat*
S10 TX assign*
S9 TX crossover
S8 TX placebo*
S7 TX random*

S6 (DE "TREATMENT OUTCOME")
S5 (DE "PLACEBOS")
S4 (DE "RESEARCH DESIGN")
S3 S2 or S1
S2 (DE "DEPRESSION") OR (DE "DEPRESSIVE DISORDER")
OR (DE "DEPRESSIVE DISORDERS")
S1 (DE "AFFECTIVE DISORDERS") OR (DE "AFFECTIVE SYMPTOMS")
