# Supplementary figures and images for: The Effects of Cognitive Therapy versus ‘No Intervention’ for Major Depressive Disorder
Source: PLoS One. 2011 Dec 9;6(12):e28299. doi: 10.1371/journal.pone.0028299 (PMC3235113; doi:10.1371/journal.pone.0028299)

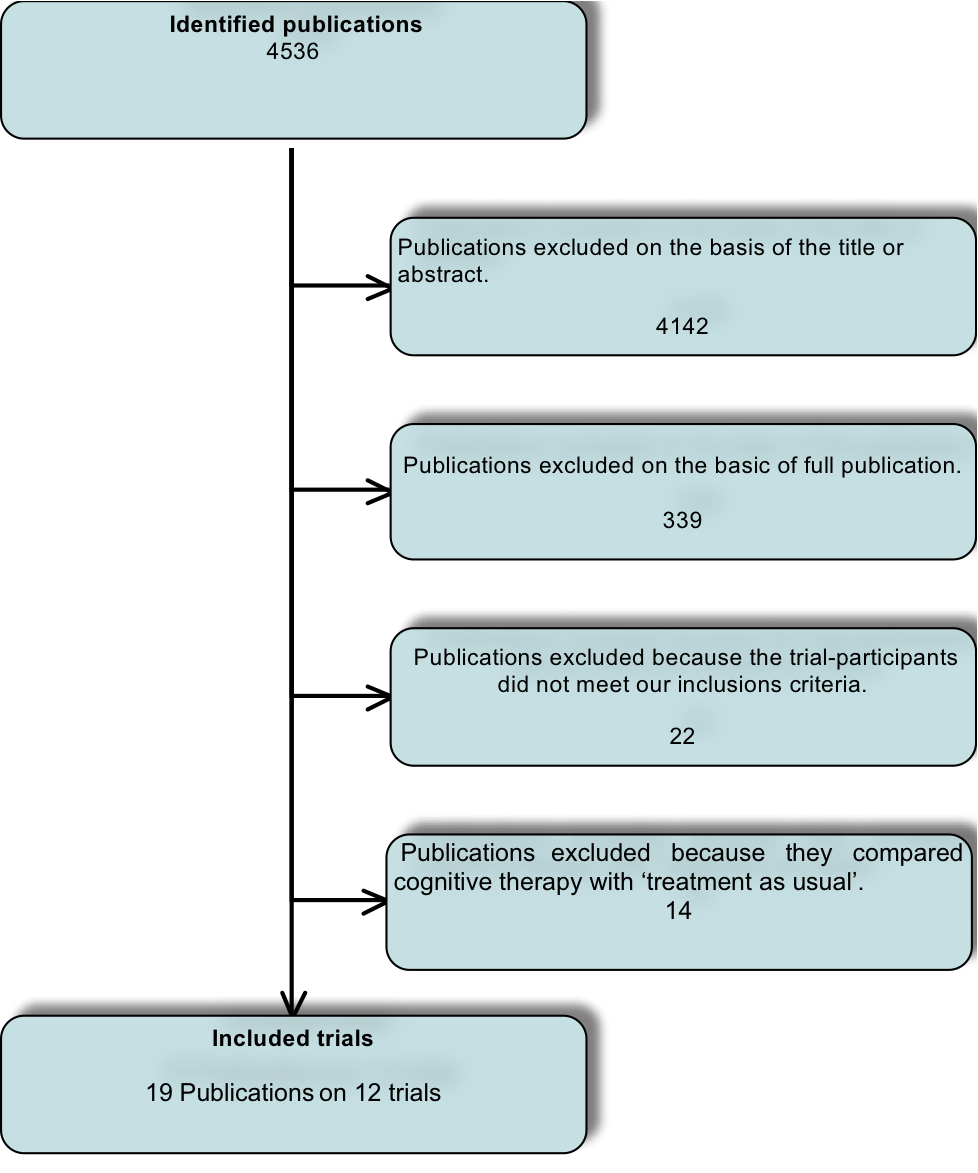

Supplement: Figure S2 — ‘PRISMA Flowchart’. (TIF) [file pone.0028299.s002.tif]
